# County-Level Social Vulnerability Is Positively Associated with Cardiometabolic Disease in Colorado

**DOI:** 10.3390/ijerph19042202

**Published:** 2022-02-15

**Authors:** Laura E. Wild, McKailey Walters, Alaina Powell, Katherine A. James, Laura Corlin, Tanya L. Alderete

**Affiliations:** 1 Department of Integrative Physiology, University of Colorado Boulder, Boulder, CO 80309, USA; laura.wild@colorado.edu (L.E.W.); alaina.powell@colorado.edu (A.P.); 2 Department of Public Health and Community Medicine, Tufts University, Boston, MA 02111, USA; mckailey.walters@tufts.edu (M.W.); laura.corlin@tufts.edu (L.C.); 3 Department Environmental and Occupational Health, University of Colorado Anschutz Medical Campus, Denver, CO 80045, USA; kathy.james@cuanschutz.edu; 4 Department of Civil and Environmental Engineering, Tufts University, Medford, MA 02155, USA

**Keywords:** the social vulnerability index, cardiometabolic disease, rural communities, rural health, Colorado, San Luis Valley

## Abstract

Cardiometabolic diseases are a group of interrelated diseases that pose greater burden among socially vulnerable communities. The social vulnerability index (SVI) identifies communities vulnerable to emergencies and may also help determine communities at risk of adverse chronic health outcomes. However, no studies have examined the relationship between the SVI and cardiometabolic health outcomes in Colorado or focused on rural settings. The aim of this ecological study was to determine whether the county-level SVI is associated with county-level cardiometabolic health indicators with a particular focus on rurality and racial/ethnic diversity. We obtained 2014 SVI scores from the Centers for Disease Control and Prevention (scored 0–1; higher = more vulnerable) and 2013–2015 cardiometabolic health estimates from the Colorado Department of Public Health and Environment. The distribution of social determinants of health was spatially evaluated. Bivariate relationships between the SVI and cardiometabolic indicators were estimated using simple linear regression models. The highest SVI scores were observed in rural areas, including the San Luis Valley (mean: 0.78, median: 0.91), Southeast (mean: 0.72, median: 0.73), and Northeast (mean: 0.66, median: 0.76) regions. Across Colorado, the SVI accounted for 41% of the variability in overweight and obesity prevalence (*p* < 0.001), 17% of the variability in diabetes prevalence (*p* = 0.001), and 58% of the age-adjusted myocardial infarction hospitalization rate (*p* < 0.001). SVI values may be useful in determining a community’s burden of cardiometabolic diseases.

## 1. Introduction

Cardiometabolic disorders are a group of interrelated health conditions including cardiovascular disease, type 2 diabetes, and obesity. Approximately 47 million Americans have at least one cardiometabolic disorder [1], and the prevalence of adverse cardiometabolic conditions is increasing [2,3]. Cardiometabolic conditions disproportionally affect vulnerable communities including low-income [4,5,6], rural [7,8], and minoritized populations [4,9,10,11,12]. For example, individuals living in rural communities are 8.6% more likely to have diabetes and 38.8% more likely to have cardiovascular disease than individuals in urban communities [8]. Furthermore, marginalized and minoritized populations within rural regions may experience even greater cardiometabolic disease burden [13].

Marginalized and minoritized communities may be particularly vulnerable to emergencies such as disease epidemics and natural or human-made disasters. One established indicator of vulnerability to these types of emergencies is the social vulnerability index (SVI) [14]. The SVI is a derived community vulnerability score that uses 15 indicators including community-level socioeconomic status (SES), vehicle ownership, household composition and housing type, prevalence of people identifying as having a disability, prevalence of people identifying as part of a racial/ethnic minority group, and linguistic diversity. Greater SVI scores reflect increased social vulnerability in terms of communities with higher poverty, greater population density, lower vehicle ownership, and fewer resources for/greater structural disparities related to racial, ethnic, and linguistic diversity [15]. Critically, having a more diverse population is not a factor that drives social vulnerability; rather, the associated structural barriers, such as institutional racism and discrimination, contribute to increased vulnerability. Although the SVI was developed for disaster preparedness, recent studies suggest that the SVI may also be helpful in identifying community-level risk of developing adverse chronic health outcomes, especially because of its characterization of several social determinants of health [16,17,18]. For example, three studies that examined the association between the SVI and cardiometabolic disease burden indicate that more socially vulnerable communities have an increased prevalence of people being overweight and obese [19,20] as well as increased prevalence of multimorbidity [21]. However, no studies have examined rural disparities in the relationship between the SVI and cardiometabolic disease burden.

Therefore, the primary aim of this ecological study was to examine the association between the county-level SVI and the county-level burden of cardiometabolic disease indicators in Colorado. We focused on rural communities due to the high burden of chronic diseases in these regions [22,23,24]. As a secondary aim, we sought to examine how racial and ethnic diversity contribute to cardiometabolic disease burden by comparing two rural areas, the San Luis Valley (SLV) and the Northeast region, that differed in terms of racial/ethnic diversity. We hypothesized that SVI scores would be positively associated with the occurrence of cardiometabolic disease and that rural communities with more racial/ethnic diversity would face a higher burden.

## 2. Materials and Methods

### 2.1. The Social Vulnerability Index (SVI)

In this ecological study, we examined county-level SVI scores for the 64 counties in Colorado. SVI scores were from the 2014 Centers for Disease Control and Prevention (CDC)/Agency for Toxic Substances and Disease Registry (ATSTDR) SVI database [25]. The 2014 SVI values were calculated from five-year estimates from the 2010–2014 American Community Survey (ACS) [26,27] and were chosen to depict the social conditions in the time frame of the selected health indicators (i.e., 2013–2015). Scores were between 0 and 1, with higher values indicating greater social vulnerability. The methods for calculating the county-level SVI from the percentile ranking of each of the 15 indicators [28] as well as the definitions of each variable [27] were previously described. The 15 indicators include poverty (% of the non-institutionalized population with total family income in the last 12 months below the family federal poverty level), unemployment (% of civilians aged 16 years or older not at work during the reference week and actively seeking work), mean per capita annual income for individuals aged 15 years old or older within a county, proportion of the population aged 25 years or older with less than a high school diploma level of education, proportion of population aged 65 or older, proportion of population aged 17 and younger, proportion of the non-institutionalized population aged five years or older who identify as having a physical or cognitive disability, proportion of households with children in single-parent households, proportion of the population who identify as a race/ethnicity other than non-Hispanic white, proportion of the population aged five years or older who identify as speaking English less than well, proportion of housing units within multi-unit structures (i.e., buildings with 10 or more housing units), proportion of housing units within mobile homes, proportion of occupied housing units with more people than rooms available, proportion of households with no vehicle available for use by household members, and proportion of the total population residing in group quarters such as correctional facilities, skilled nursing facilities, and college residence halls [14,27].

### 2.2. Health Indicators

We obtained three-year county-level data for from the 2013–2015 Colorado Department of Public Health and Environment (CDPHE) Colorado Health Indicators (Version 6.0) [29]. Health indicator definitions came from the CDPHE and include the percent of adults aged 18 years and older with overweight or obesity (i.e., body mass index [BMI] greater than 25 kg/m^2^), obesity (i.e., BMI greater than or equal to 30 kg/m^2^), diabetes (diagnosed by a health care provider), and elevated blood pressure (diagnosed by a health care provider). Other health indicators included the age-adjusted rate of heart disease hospitalizations per 100,000 population, age-adjusted rate of acute myocardial infarction hospitalizations per 100,000 population, and the percent of live births to mothers who had overweight or obesity prior to pregnancy (i.e., pre-pregnancy overweight and obesity).

### 2.3. Geographic Regions

Colorado counties were separated into 10 regions determined by the Colorado Department of Local Affairs [30]. Although our primary analysis was conducted at the county level, we also identified two specific rural regions of interest given the high burden of chronic disease in rural areas [22,23,24,31]. The first was the SLV, which includes Alamosa, Conejos, Costilla, Mineral, Rio Grande, and Saguache counties. The second was the Northeast Region, including Kit Carson, Logan, Morgan, Phillips, Sedgwick, Washington, and Yuma counties. These rural regions differed in their racial/ethnic composition; nearly 45% of the SLV identified as a race/ethnicity other than non-Hispanic white whereas only 24% of the Northeast identified as a race/ethnicity other than non-Hispanic white. The other eight geographic regions across Colorado included the Central Mountains Region (i.e., Chaffee, Custer, El Paso, Fremont, Park, Pueblo, and Teller counties), Greater Metro Region (i.e., Adams, Arapahoe, Broomfield, Clear Creek, Denver, Douglas, Elbert, Gilpin, Jefferson, Lincoln counties), I-70 West Region (i.e., Eagle, Garfield, Lake, Pitkin, Rio Blanco, and Summit counties), North Central Region (i.e., Boulder, Jackson, Larimer, and Weld counties), Northwest Region (i.e., Grand, Moffat, and Routt counties), Southeast Region (i.e., Baca, Bent, Cheyenne, Crowley, Huerfano, Kiowa, Las Animas, Otero, and Prowers counties), Southwest Region (i.e., Archuleta, Dolores, La Plata, Montezuma, and San Juan counties), and West Central Region (i.e., Delta, Gunnison, Hinsdale, Mesa, Montrose, Ouray, and San Miguel counties).

### 2.4. Analytic Methods

We used a Geographic Information System (ArcMap 10.8.1) to generate maps showing the spatial distribution of SVI indicators as well as the overall SVI score [25]. We also created maps to demonstrate the spatial distribution of health care facilities (i.e., hospitals, clinics, and federally qualified health clinics [FQHC]) within the SLV, Northeast region, and Greater Metro region to examine the differences in rural and urban settings for these resources (Supplemental Figure S1). In addition, we used simple linear regression to examine bivariate relationships between the county-level SVI and each county-level cardiometabolic health indicator. We examined the adjusted R^2^ as a measure of the strength of association, and the *p*-value as an indicator of statistical significance (*p* < 0.05 was considered statistically significant). Secondary analyses fit simple linear regression models for counties just in the SLV and just in the Northeast region. Statistical analyses were conducted with Stata SE version 16.1. This study was considered not human subjects research by the Tufts Health Sciences Institutional Review Board (study 00002372).

## 3. Results

### Spatial Patterning of Social Vulnerability across Colorado

**Figure 1:**
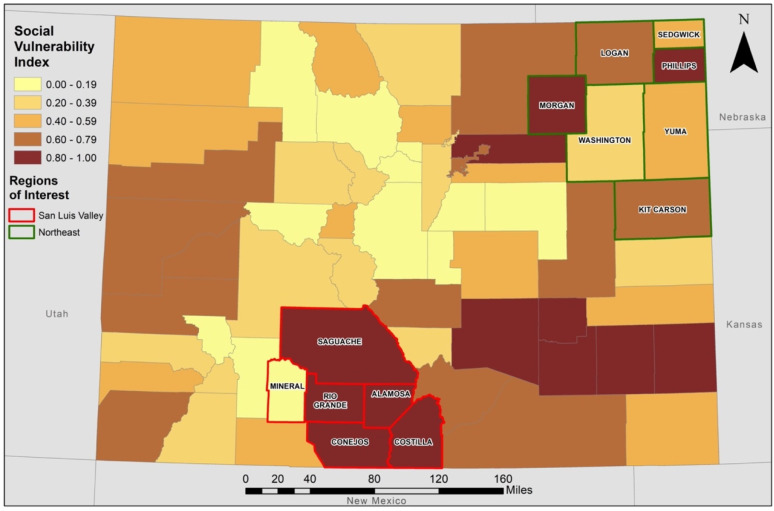
Social Vulnerability Index Scores for Counties in Colorado. SVI scores were determined for each county in Colorado from 15 social variables from the 2014 Centers for Disease Control and Prevention (CDC)/Agency for Toxic Substances and Disease Registry (ATSTDR) database. Higher scores indicate higher vulnerability. Counties in the San Luis Valley are outlined in red and counties in the Northeast region are outlined in green.

As shown in Table 1 and Figure 1, county-level SVI scores in Colorado were between 0.0 and 1.0, with the counties with the 10 highest scores all in the rural SLV, Southeast, and Northeast regions. Within the SLV, five of the six counties (i.e., Alamosa, Conejos, Costilla, Rio Grande, and Saguache) had SVI scores between 0.83 and 0.97 (mean for all six counties: 0.78, median for all six counties: 0.91). Only one county within the SLV, Mineral County, had low social vulnerability as indicated by an SVI score of 0.08. In contrast, the SVI scores in the rural Northeast region were between 0.33 and 0.92 (mean for all seven counties: 0.66, median for all seven counties: 0.76).

**Figure 2:**
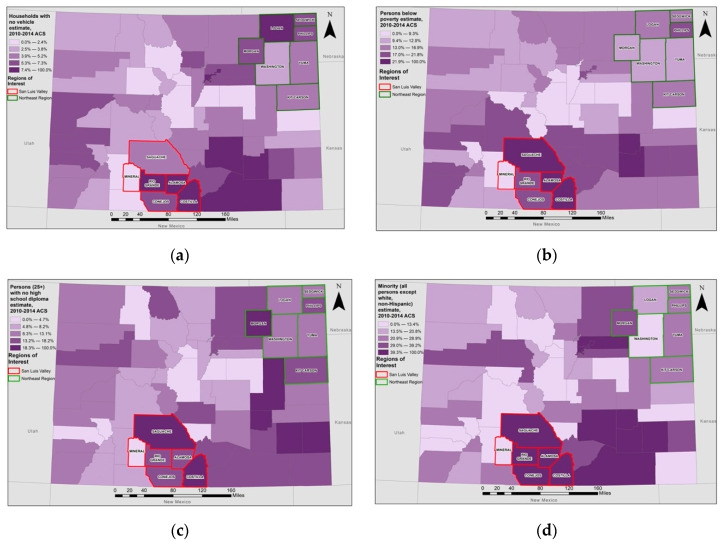
Social Vulnerability Index Indicators by County Across Colorado. County-level spatial distribution of the (**a**) proportion of households without access to vehicles, (**b**) proportion of the population with total family income below the family federal poverty level, (**c**) proportion of the adult population without a high school diploma, and (**d**) proportion of the population who identify as a race/ethnicity other than non-Hispanic white. Threshold values reflect natural breaks in the data. Counties in the San Luis Valley are outlined in red and counties in the Northeast region are outlined in green. Data come from the 2014 Centers for Disease Control and Prevention (CDC)/Agency for Toxic Substances and Disease Registry (ATSTDR) database.

As shown in Table 2 and Figure 2, factors contributing to the high SVI scores in the SLV were limited access to vehicles (6.6% of households), high poverty (20.6% of population), low educational attainment (16.2% of population had no high school diploma), and a large minoritized population (44.9% of population). In contrast, the Northeast region had less limited access to vehicles (5.9% of households), lower poverty (14.6% of population), higher educational attainment (14.1% of population had no high school diploma), and a smaller minoritized population (24.3% of population).

**Figure 3:**
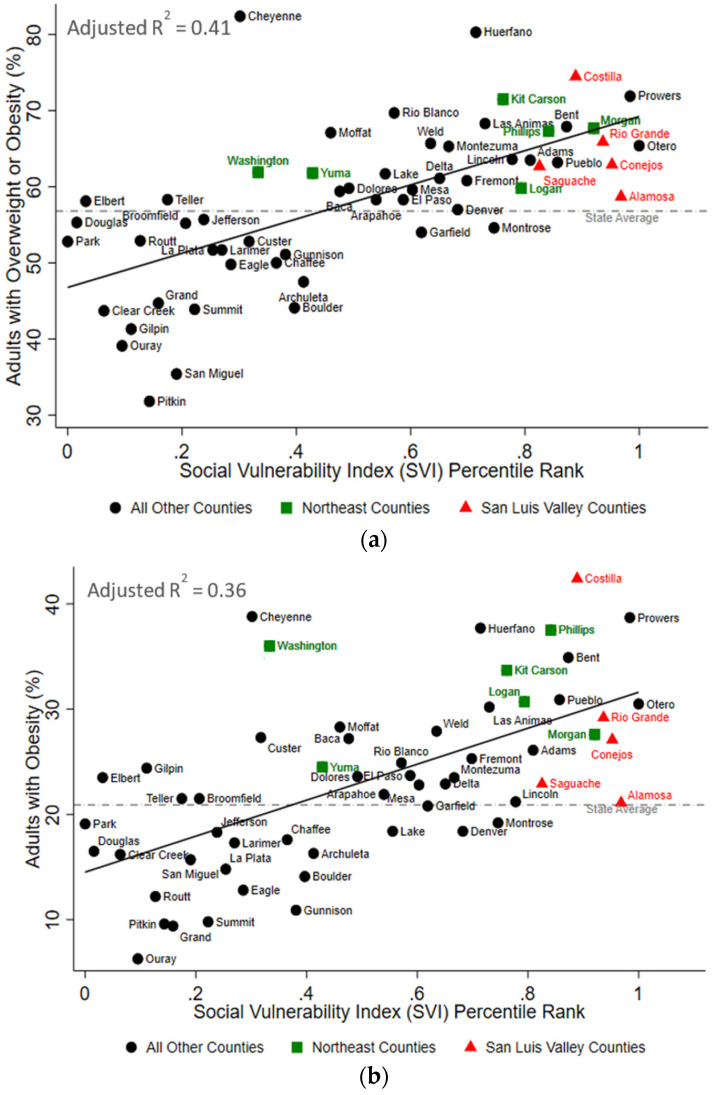

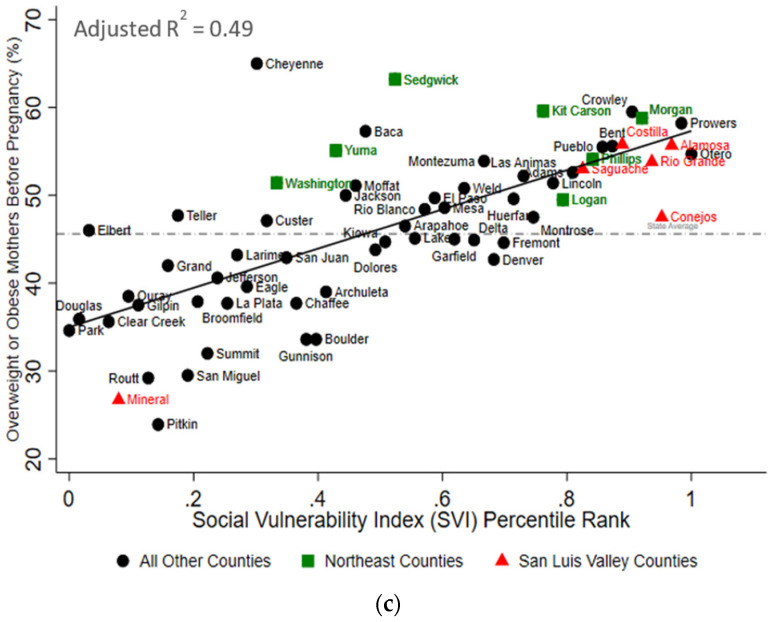
The Social Vulnerability Index is Positively Associated with Overweight and Obesity in Colorado. In bivariate analyses, the county-level social vulnerability index was statistically associated with the percent of adults with (**a**) overweight or obesity, (**b**) obesity, and (**c**) overweight or obesity prior to pregnancy. The red triangles represent counties in the San Luis Valley region of Colorado, the green squares represent counties in the Northeast region of Colorado, and the black circles represent all other counties in Colorado. Information regarding adults with overweight and obesity and obesity were not available for Mineral County. The dashed grey lines represent the state average, and the solid black lines represent the respective lines of best fit. R^2^ values are adjusted R^2^.

**Figure 4:**
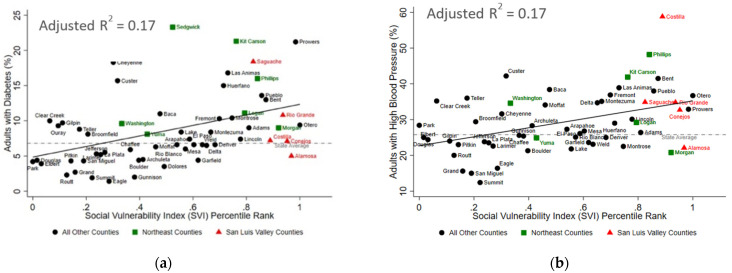

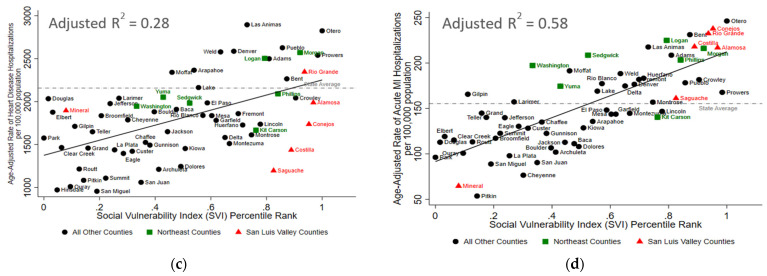
The Social Vulnerability Index is Positively Associated with Adverse Cardiometabolic Outcomes in Colorado. In bivariate analyses, the county-level social vulnerability index was statistically associated with (**a**) the percent of adults with diabetes, (**b**) the percent of adults with high blood pressure, (**c**) age-adjusted rate of heart disease hospitalizations (per 100,000 people), and (**d**) the age-adjusted rate of acute myocardial infarction (MI) hospitalizations (per 100,000 people). The red triangles represent counties in the San Luis Valley region of Colorado, the green squares represent counties in the Northeast region of Colorado, and the black circles represent all other counties in Colorado. Information regarding adults with obesity and high blood pressure were not available for Mineral County. The dashed grey lines represent the state average, and the solid black lines represent the respective lines of best fit. R^2^ values are adjusted R^2^.

The SLV region had between 11 and 38% higher values for each of the cardiometabolic health indicators compared to the state average except for the age-adjusted rate of hospitalizations due to heart disease, which was 13% below the state average (Table 3). Similarly, the Northeast region had between 5 and 53% higher values for each of the cardiometabolic health indicators compared to the state average (Table 3). Across Colorado, the higher county-level SVI was significantly associated with a higher burden of cardiometabolic outcomes in Colorado (Figure 3 and Figure 4). The SVI accounted for 41% (*p* < 0.001), 36% (*p* < 0.001), and 49% (*p* < 0.001) of the variability in adult overweight and obesity, obesity, and pre-pregnancy overweight or obesity outcomes, respectively. Similarly, the SVI accounted for 17% of the variability in diabetes prevalence (*p* = 0.001), 17% of the variability in high blood pressure prevalence (*p* = 0.001), 28% of the variability in age-adjusted rate of hospitalizations due to heart disease (*p* < 0.001), and 58% of the variability in age-adjusted rate of hospitalizations due to myocardial infarctions (*p* < 0.001). Finally, as shown in Table 4, restricting these bivariate analyses to only counties from the SLV or only counties from the Northeast region demonstrated that the amount of variability explained by the SVI was greater in the SLV, than in the Northeast for three of the cardiometabolic outcomes (i.e., pre-pregnancy overweight and obesity, diabetes, and acute myocardial infarction hospitalizations). Importantly, these analyses were under-powered (based on the number of counties per region and the limited variability in the SVI within these regions).

## 4. Discussion

This ecologic study is the first known to assess the association between county-level SVI scores and cardiometabolic outcomes across Colorado. We observed significant positive associations with each cardiometabolic indicator presented. Additionally, our study is the first of which we are aware that used the SVI in Colorado with a focus on rural regions and areas with large minoritized populations. Our study supports the body of evidence indicating that the burden of cardiometabolic disease is higher among rural [7,8] and minoritized populations [4,9,10,11,12]. It also adds to a growing body of literature suggesting that the SVI may be useful in identifying communities vulnerable to adverse chronic health outcomes. This finding agrees with prior research indicating that several of the SVI indicators are also risk factors for poor cardiometabolic health outcomes (e.g., limited access to transportation [32], poverty [33,34,35,36], and limited educational attainment [37,38,39]).

We focused primarily on two rural regions with a high burden of chronic disease [22,23,24]. In the SLV region, there is a large proportion of the community that identified as non-Hispanic white, and four of the six counties had SVI scores among the top 10 for counties across Colorado. One of the drivers of the high SVI in this rural region was the high proportion of the population without access to a vehicle. This structural barrier, coupled with the far distance (often up to 120 miles) to specialty medical and surgical care [40] may contribute to the high cardiometabolic disease burden observed in this region. Similarly, the high poverty burden observed in the rural SLV region may contribute to other cardiometabolic risk factors such as food insecurity [41] and less access to recreational physical activity [42].

The Northeast region was the second rural area of interest in our study and the SVI scores were generally lower in this region than for most of the SLV counties. The Northeast region had only about half the percentage of its population that identified as non-Hispanic white as the percentage in the SLV. Although the burden of cardiometabolic disease was high in both rural regions, we observed some evidence that the associations between the SVI and cardiometabolic health were less robust in the Northeast region than in the SLV region for three of the six cardiometabolic outcomes we examined. This trend could indicate that maintaining cardiometabolic health may be more challenging in the more racially/ethnically diverse SLV region than in the less diverse Northeast region. If true, it is possible that structural discrimination and racism may further limit access to important resources in these rural areas (e.g., transportation, education, and healthcare) that contribute to cardiometabolic health disparities [43,44,45]. However, future studies are needed and should aim to replicate these rural specific findings since comparisons between these regions were based on a limited sample size (i.e., 5–7 data points) and findings were not consistent across each of the health indicators included in our study.

Our results add to a growing body of research that suggests the SVI may be a useful tool in identifying vulnerable communities at risk of adverse health outcomes such as overweight and obesity [19,20], multimorbidity among those with obesity and cardiovascular diseases [21], as well as COVID-19 incidence [46,47] and COVID-19 mortality [47]. Additionally, our findings are consistent with national health disparity trends and highlight the need for individual, family, and community levels efforts to ensure health equity in marginalized and minoritized communities. Finally, it is important to note that other indices of social vulnerability (e.g., CalEnvironScreen, California Healthy Places Index, and structural racism index) have been associated with adverse health outcomes [48,49,50,51,52]. Although we chose the SVI in this study due to data availability, future studies using other similar composite vulnerability indicators would be expected to observe similar results. Future studies could consider targeting the individual components that make up these indices of social vulnerability to inform existing and future prevention and intervention strategies at the community level. For example, this may include increasing access to public transportation through local (e.g., Valley-Wide Ride in SLV), state (e.g., Colorado Rural Health Center), and federal programs (e.g., National Rural Transit Assistance Program and Community Transportation Association of America) to reduce adverse health outcomes. In addition, established county-level collaborations within Colorado, such as the San Luis Valley Public Health Partnership, work to reduce disparities in health through collaborative efforts across systems to increase access to healthy food and reduce environmental exposures, injuries, and drug use; however, these efforts have significantly decreased due to the COVID-19 pandemic.

To our knowledge, this is the first study to show that greater social vulnerability is associated with adverse cardiometabolic outcomes in a rural setting with an emphasis on the intersection between rurality and race/ethnicity. Nevertheless, our study had several limitations. The 15 indicators that are used to calculate the SVI are often correlated [53], making it difficult to determine which indicators are most influential in the observed associations with cardiometabolic health burden. Second, information regarding prevalence of cardiometabolic indicators for Mineral County was limited. Similarly, for all of Colorado, we lacked information needed to examine potential confounders such as the variability in resources (e.g., health care facilities, recreational facilities, and other community support) beyond what is being captured by the SVI. Lastly, since we examined bivariate associations, our findings may introduce an ecological fallacy. However, many population-level covariates were already addressed within the 15 indicators comprising the SVI score. Nevertheless, future studies should examine individual-level risk factors, confounders, and outcomes in the context of community-level vulnerability.

## 5. Conclusions

Our study indicates that socially vulnerable communities have an increased burden of adverse cardiometabolic health outcomes, and these associations may be exacerbated by rurality and racial/ethnic disparities. These results highlight the importance of understanding and addressing social determinants of health with intervention strategies at the local, state, and federal levels. Furthermore, our study supports the use of the SVI in future larger-scale, longitudinal research studies assessing how trends vary over time. In the long term, the SVI may be useful in practical and applied settings to identify and mitigate community-level risk for adverse chronic health outcomes.

## Figures and Tables

**Table 1:** County-Level Social Vulnerability Index Scores.

County	SVI Score	County	SVI Score
Central Mountains Region		Northwest Region	
Chaffee	0.37	Grand	0.16
Custer	0.32	Moffat	0.46
El Paso	0.59	Routt	0.13
Fremont	0.70	San Luis Valley	
Park	0.00	Alamosa	0.97
Pueblo	0.86	Conejos	0.95
Teller	0.17	Costilla	0.89
Greater Metro Region		Mineral	0.08
Adams	0.81	Rio Grande	0.94
Arapahoe	0.54	Saguache	0.83
Broomfield	0.21	Southeast Region	
Clear Creek	0.06	Baca	0.48
Denver	0.68	Bent	0.87
Douglas	0.02	Cheyenne	0.30
Elbert	0.03	Crowley	0.90
Gilpin	0.11	Huerfano	0.71
Jefferson	0.24	Kiowa	0.51
Lincoln	0.78	Las Animas	0.73
I-70 West Region		Otero	1.00
Eagle	0.29	Prowers	0.98
Garfield	0.62	Southwest Region	
Lake	0.56	Archuleta	0.41
Pitkin	0.14	Dolores	0.49
Rio Blanco	0.57	La Plata	0.25
Summit	0.22	Montezuma	0.67
North Central Region		San Juan	0.35
Boulder	0.40	West Central Region	
Jackson	0.44	Delta	0.65
Larimer	0.27	Gunnison	0.38
Weld	0.63	Hinsdale	0.05
Northeast Region		Mesa	0.60
Kit Carson	0.76	Montrose	0.75
Logan	0.79	Ouray	0.10
Morgan	0.92	San Miguel	0.19
Phillips	0.84		
Sedgwick	0.52		
Washington	0.33		
Yuma	0.43		

County-level SVI scores were calculated from 15 social variables from the 2014 Centers for Disease Control and Prevention (CDC)/Agency for Toxic Substances and Disease Registry (ATSTDR) database. SVI scores are calculated with a range of 0.0–1.0 with higher scores indicating greater social vulnerability. Regions were determined by the Colorado Department of Local Affairs.

**Table 2:** Social Vulnerability Index Indicator Values in the San Luis Valley and Northeast Regions of Colorado.

	Below Poverty (%)	Unemployed (%)	Per Capita Income ($)	No High School Diploma (%)	Aged 65 and Older (%)	Aged 17 or Younger (%)	Older than Age 5 with a Disability (%)	Single-Parent Households (%)	Minority (%)	Speaks English “Less Than Well” (%)	Multi-unit Structures (%)	Mobile Homes (%)	Crowding (%)	No Vehicle (%)	Group Quarters (%)
**Counties**															
San Luis Valley	20.6	9.3	22,383	16.2	20.8	20.8	20.0	6.6	44.9	3.0	1.9	16.1	2.6	6.6	2.6
Alamosa	27.9	10.7	19,481	15.4	12.0	24.3	15.8	9.3	50.5	2.8	5.1	17.1	2.7	8.8	7.0
Conejos	18.6	9.2	18,247	16.0	16.4	27.7	19.7	8.9	56.9	3.1	1.4	19.4	3.8	5.9	0.9
Costilla	25.0	15.1	20,592	24.5	24.2	20.3	30.4	7.3	68.5	2.8	0.2	18.5	1.5	9.4	0.0
Mineral	6.8	0.6	34,305	3.5	38.9	5.8	19.7	0.0	4.3	0.3	0.0	10.1	0.0	1.6	4.5
Rio Grande	20.1	8.8	21,104	16.2	17.1	24.5	16.3	8.0	46.3	2.9	4.1	14.0	3.7	9.0	2.3
Saguache	25.1	11.4	20,569	21.5	15.9	22.3	17.9	6.0	43.1	5.8	0.7	17.4	3.7	4.6	0.7
Northeast Region	14.6	6.6	22,644	14.1	18.2	23.3	13.4	8.3	24.3	4.1	3.8	10.4	2.5	5.9	3.9
Kit Carson	15.5	6.5	21,330	16.8	17.4	22.4	16.9	10.4	24.9	3.2	4.2	14.6	1.3	4.0	10.1
Logan	16.9	11.8	23,533	12.4	15.4	19.2	14.4	7.9	20.2	2.8	5.8	12.0	2.7	9.0	4.8
Morgan	12.3	5.8	21,297	20.8	14.7	27.3	11.5	12.7	39.2	8.3	6.7	14.5	5.3	7.0	2.1
Phillips	18.6	6.1	22,996	16.2	19.6	25.6	13.9	8.1	28.2	7.3	2.9	10.0	5.1	6.6	1.6
Sedgwick	15.6	8.0	22,124	12.1	23.7	20.0	14.3	8.8	23.1	0.2	2.6	5.8	0.8	6.1	1.8
Washington	12.9	5.5	24,326	9.5	20.1	21.7	12.6	6.0	11.4	1.2	1.3	7.8	1.4	3.4	5.3
Yuma	10.2	2.6	22,902	11.1	16.7	26.8	10.3	4.4	22.9	5.4	3.0	8.1	1.0	5.0	1.4

Values were obtained from the 2010–2014 American Community Survey and represent the frequency (%) and mean (for per capita income) of the 15 variables used to calculate the county-level SVI scores. Data come from the 2014 Centers for Disease Control and Prevention (CDC)/Agency for Toxic Substances and Disease Registry (ATSTDR) database. Regional values are the unweighted averages for all counties in the region.

**Table 3:** Prevalence of Cardiometabolic Health Indicators in the San Luis Valley and Northeast Regions of Colorado.

	Adults with Overweight or Obesity (%) [95% CI]	Adults with Obesity (%) [95% CI]	Overweight or Obese Mothers before Pregnancy (%) [95% CI]	Adults with Diabetes (%) [95% CI]	Adults with High Blood Pressure (%) [95% CI]	Age-Adjusted Rate of Heart Disease Hospitalizations per 100,000 [95% CI]	Age-Adjusted Rate of AMI Hospitalizations per 100,000 [95% CI]
**Counties**							
San Luis Valley	62.8 [57.7, 67.8]	25.6 [20.9, 30.4]	53.0 [50.5, 55.5]	8.5 [6.3, 10.7]	32.5 [26.5, 38.4]	1869.1 [1805.7, 1932.6]	214.3 [192.1, 236.6]
Alamosa	58.7 [49.1, 68.4]	21.1 [13.5, 28.8]	55.7 [51.7, 59.7]	5.0 [2.8, 7.3]	22.1 [14.3, 29.9]	1987.8 [1865.8, 2109.8]	216.9 [175.3, 258.5]
Conejos	62.9 [51.4, 74.4]	27.1 [16.4, 37.8]	47.5 [42.0, 53.0]	7.1 [2.0, 12.3]	32.7 [21.4, 44.0]	1736.5 [1594.0, 1879.1]	237.8 [182.5, 293.1]
Costilla	74.5 [61.2, 87.8]	42.4 [21.7, 63.0]	55.8 [44.8, 66.9]	7.2 [2.0, 12.3]	58.8 [32.1, 85.6]	1435.1 [1244.8, 1625.3]	218.0 [147.3, 288.8]
Mineral	–	–	26.7 [8.2, 51.0]	–	–	1894.9 [1456.3, 2333.5]	64.6 [1.8, 216.7]
Rio Grande	65.9 [56.8, 74.9]	29.2 [19.3, 39.1]	53.8 [48.8, 58.8]	10.8 [5.4, 16.3]	34.8 [24.3, 45.3]	2345.9 [2209.7, 2482.1]	232.4 [186.5, 278.3]
Saguache	62.7 [50.8, 74.7]	22.9 [12.9, 32.8]	53.0 [46.1, 59.9]	18.4 [9.2, 27.7]	34.9 [19.3, 50.5]	1195.4 [1056.6, 1334.2]	161.2 [110.6, 211.9]
Northeast Region *	64.3 [59.6, 69.0]	29.5 [25.6, 33.4]	55.4 [53.6, 57.3]	10.4 [8.2, 12.7]	27.1 [22.7, 31.4]	2363.2 [2308.2, 2418.1]	209.2 [192.2, 226.3]
Kit Carson	71.5 [63.0, 79.9]	33.7 [22.9, 44.5]	59.6 [54.0, 65.2]	21.3 [4.8, 37.9]	41.9 [25.7, 58.2]	1666.1 [1527.8, 1804.4]	140.4 [98.5, 182.4]
Logan	59.8 [51.4, 68.2]	30.7 [23.4, 38.0]	49.5 [45.7, 53.2]	11.1 [6.4, 15.7]	29.2 [20.6, 37.9]	2502.8 [2400.0, 2605.5]	224.9 [192.3, 257.4]
Morgan	67.7 [58.9, 76.4]	27.6 [21.1, 34.0]	58.8 [56.2, 61.5]	9.0 [5.7, 12.3]	20.8 [14.6, 26.9]	2571.2 [2476.6, 2665.8]	216.1 [187.3, 244.8]
Phillips	67.3 [54.9, 79.7]	37.5 [23.9, 51.0]	54.1 [46.6, 61.5]	16.0 [6.9, 25.0]	48.2 [33.6, 62.8]	2089.2 [1894.2, 2284.2]	203.3 [140.4, 266.3]
Sedgwick	–	–	63.2 [52.3, 74.0]	23.3 [6.8, 39.7]	–	1983.1 [1716.2, 2249.9]	208.6 [125.6, 291.6]
Washington	61.9 [47.3, 76.6]	36.0 [20.2, 51.8]	51.4 [43.2, 59.6]	9.6 [1.8, 17.5]	34.6 [11.1, 58.1]	1945.4 [1761.8, 2129.0]	197.3 [139.0, 255.6]
Yuma	61.8 [51.9, 71.7]	24.5 [16.9, 32.0]	55.1 [50.3, 60.0]	8.1 [3.3, 12.8]	24.9 [15.9, 34.0]	2051.9 [1917.2, 2186.5]	174.4 [133.2, 215.6]
**State Average**	56.8 [56.1, 57.5]	20.9 [20.4, 21.5]	45.6 [45.4, 45.8]	6.8 [6.5, 7.1]	25.8 [25.1, 26.5]	2156.9 [2150.0, 2163.7]	155.3 [153.4, 157.2]

Data are from the 2013–2015 Colorado Department of Public Health and Environment (CDPHE) Colorado Health Indicators (Version 6.0). * Based on available data, regional values do not include Kit Carson County. AMI represents acute myocardial infarction.

**Table 4:** Bivariate Associations between the Social Vulnerability Index (0–100) and Cardiometabolic Health Indicators among Only Counties in the San Luis Valley and Northeast Regions of Colorado.

	Adults with Overweight or Obesity	Adults with Obesity	Overweight or Obese Mothers before Pregnancy	Adults with Diabetes	Adults with High Blood Pressure	Age-Adjusted Rate of Heart Disease Hospitalizations per 100,000	Age-Adjusted Rate of AMI Hospitalizations per 100,000
San Luis Valley							
Adjusted R^2^	0.00	0.00	0.87	0.62	0.00	0.00	0.89
β	−0.31	−0.10	2.91	−0.93	−0.19	−0.003	0.49
*p*-value	0.60	0.81	0.004	0.07	0.45	0.95	0.003
Northeast Region							
Adjusted R^2^	0.10	0.00	0.00	0.00	0.00	0.08	0.00
β	2.79	0.25	0.44	0.41	0.24	0.03	0.15
*p*-value	0.28	0.92	0.84	0.81	0.85	0.28	0.68

Simple linear regression examined the bivariate relationships between the county-level SVI and each county-level cardiometabolic health indicator for the San Luis Valley and Northeast regions. We examined the adjusted R^2^ as a measure of the strength of association and the *p*-value as an indicator of statistical significance. Due to their small size, β are presented on a 0–100 scale for easier interpretability. AMI represents acute myocardial infarction.

## Data Availability

Publicly available datasets were analyzed in this study. This data can be found here: https://www.atsdr.cdc.gov/placeandhealth/svi/data_documentation_download.html (accessed on 31 December 2021) and https://cdphe.colorado.gov/workplace-safety/data-and-reports/colorado-health-indicators (accessed on 31 December 2021).

## Supplementary Materials

The following supporting information can be downloaded at: https://www.mdpi.com/article/10.3390/ijerph19042202/s1, Figure S1: Spatial distribution of health care facilities within the San Luis Valley, Northeast region, and Greater Metro Area in Colorado (County-level spatial distribution of the health care facilities for (a) San Luis Valley (SLV), (b) Northeast region, and (c) Greater Metro region. Health care facilities were classified as hospitals, clinics, and federally qualified health clinics (FQHC). Health facility data were obtained from the Colorado Department of Public Health and Education (CDPHE)).
