# Supplementary material for: County-Level Social Vulnerability Is Positively Associated with Cardiometabolic Disease in Colorado
Source: Int J Environ Res Public Health. 2022 Feb 15;19(4):2202. doi: 10.3390/ijerph19042202 (PMC8872484; doi:10.3390/ijerph19042202)
Supplement: Supplementary file 1 [file ijerph-19-02202-s001.zip › ijerph-1561714-supplementary.pdf]

Supplemental Figure S1. Spatial distribution of health care facilities within the San Luis Valley, Northeast region, and Greater Metro Area in Colorado

(a) (b) (c)

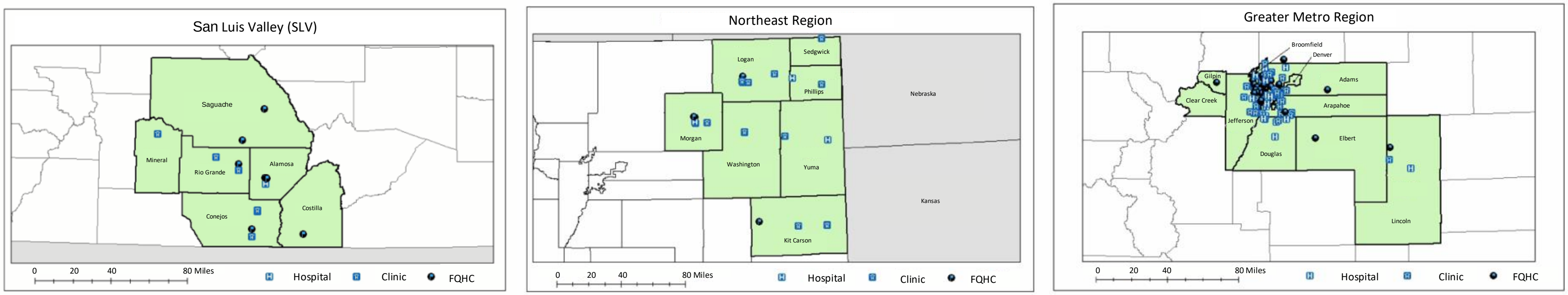

Supplemental Figure S1. County-level spatial distribution of the health care facilities for (a) San Luis Valley (SLV), (b) Northeast region, and (c) Greater Metro region. Health care facilities were classified as hospitals, clinics, and federally qualified health clinics (FQHC). Health facility data were obtained from the Colorado Department of Public Health and Education (CDPHE).
